# Surface Development of Polyethylene Terephthalate Films Using Low-Pressure, High-Frequency Argon + Oxygen Plasma on Zinc Powder for Dye-Sensitized Solar Cells

**DOI:** 10.3390/polym16162283

**Published:** 2024-08-12

**Authors:** Wittawat Poonthong, Narong Mungkung, Khanchai Tunlasakun, Nuttee Thungsuk, Nat Kasayapanand, Somchai Arunrungrusmi, Tanes Tanitteerapan, Threerapong Maneepen, Apidat Songruk, Toshifumi Yuji

**Affiliations:** 1 School of Energy Environment and Materials, King Mongkut’s University of Technology Thonburi, Bangkok 10140, Thailand; wittawat.poon@kmutt.ac.th (W.P.); narong.mun@kmutt.ac.th (N.M.); 2 Faculty of Industrial Education and Technology, King Mongkut’s University of Technology Thonburi, Bangkok 10140, Thailand; khanchai.tun@kmutt.ac.th (K.T.); tanes_kmutt@yahoo.com (T.T.); theerapong.man@kmutt.ac.th (T.M.); apidat.song@kmutt.ac.th (A.S.); 3 Department of Electrical Engineering, Dhonburi Rajabhat University Samut-Prakan, Samut-Prakan 10150, Thailand; nuttee.t@dru.ac.th; 4 Faculty of Education, University of Miyazaki, Miyazaki 889-2192, Japan; yuji@cc.miyazaki-u.ac.jp

**Keywords:** PET films, zinc powder, Ar + O_2_ plasma, RF power, LPCVD, DSSCs

## Abstract

This research has developed a process for producing ZnO thin film from DEZn deposited onto a PET substrate with low-pressure, high-frequency Ar + O_2_ plasma using a chemical vapor deposition technique. The aim is to study the film production conditions that affect electrical properties, optical properties, and thin film surfaces. This work highlights the use of plasma energy produced from a mixture of gases between Ar + O_2_. Plasma production is stimulated by an RF power supply to deliver high chemical energy and push ZnO atoms from the cathode inside the reactor onto the substrate through surface chemical reactions. The results showed that increasing the RF power in plasma production affected the chemical reactions on the substrate surface of film formations. Film preparation at an RF power of 300 W will result in the thickest films. The film has a continuous columnar formation, and the surface has a granular structure. This results in the lowest electrical resistivity of 1.8 × 10^−4^ Ω. In addition, when fabricated into a DSSC device, the device tested the PCE value and showed the highest value at 5.68%. The reason is due to the very rough surface nature of the ZnO film, which increases the scattering and storage of sunlight, making cells more efficient. Therefore, the benefit of this research is that it will be a highly efficient prototype of thin film production technology using a chemical process that reduces production costs and can be used in the industrial development of solar cells.

## 1. Introduction

Solar cell technology is a suitable renewable energy source to complement the increase in the world’s consumption, which has a minimal environmental impact [1]. For many years, there has been a growing interest in developing low-cost solar cells using molecular materials and semiconductor nanostructures. Silicon solar cells dominate the commercial market, but production processes involve high energy and vacuum conditions, leading to high production costs. For these reasons, dye-sensitized solar cells (DSSCs) and organic solar cells (OSCs) [2,3,4,5] show their potential as relatively low-cost alternatives to conventional solar cell silicon. The exciting advantage of this type of solar cell is the potential for roll-to-roll printing on polymer substrates [6,7]. The absence of liquid electrolytes also makes the processing and sealing of solar cell devices relatively easier. With the advancement of technology, there is a need for lightweight and flexible electronic devices. The advantage is that they can be produced on a large scale using room-temperature techniques with significantly reduced costs. Therefore, the idea of flexible polymer-based solar cells has attracted great interest among many researchers in the DSSC field. In the solar energy industry, polyethylene terephthalate (PET) film is one of the most critical strategic materials due to its excellent mechanical strength, reflectivity, water resistance, and mainly mechanical and insulating properties [8,9,10,11]. PET is a thin film created from polyester stretched biaxially. It can withstand temperatures as low as −70 degrees and over 200 degrees. PET films must meet various performance criteria, including high transparency, minimal turbidity, and high brightness. These factors are essential challenges in the development of this field. In solar cells, a metal oxide film such as titanium dioxide (TiO_2_) [12] or zinc oxide (ZnO) [13,14,15] is often used as an electron transport layer to transfer electrons and block holes beneficially. The metal oxide layer can be considered a modification agent to reduce the functionality of the modified anode of solar cell devices [16,17,18]. It is well known that the presence of oxygen vacancies in ZnO can increase the charge density of ZnO to form an n-type semiconductor [19,20,21], which is an excellent property to make ZnO more stable. The advantages include good transparency and high electron mobility, producing good electrical conductivity. ZnO is a semiconductor with a wide band gap of approximately 3.37 electron volts (eV). Its crystal structure is a hexagonal closed pack (HCP). Inside the structure, oxygen ions (O^2-^) are inserted in the middle position between zinc ions (Zn^2+)^ [22,23,24]. Thin films can be prepared using various methods, among which chemical vapor deposition (CVD) has been widely accepted [25,26,27]. CVD involves the formation of a solid film on a substrate by a chemical reaction in the gas or vapor phase. The process uses a variety of gas, liquid, and solid chemicals as the source of components used to create thin films. Compared with most thin film preparation methods, CVD has several unique advantages, such as adaptability, productivity, versatility, quality, and reproducibility. For these reasons, CVD continues to expand and evolve into the most important method for producing films for solid-state devices. The main focus during the development of CVD films is film preparation techniques. The CVD films are preliminarily prepared using conventional methods at atmospheric pressure, such as low-pressure chemical vapor deposition (LPCVD) [28], spray pyrolysis chemical vapor deposition (SPCVD) [29], metal–organic chemical vapor deposition (MOCVD) [30], and plasma-enhanced chemical vapor deposition (PECVD) [31]. More specifically, LPCVD deposits a thin film on the substrate heated to high or low temperatures in a reactor under reduced pressure. This method has the following advantages: improving film thickness and composition uniformity, controlling the deposition rate with only the surface reaction, reducing the number of defects, improving suitability, and covering large-scale production processes [32,33]. However, many issues related to the fabrication of flexible DSSCs still need to be addressed. This study reports on the technological development of DSSCs on flexible surfaces. It focuses on PET surfaces and factors related to film deposition and electrode processing to improve the devices’ mechanical and solar cell properties.

Therefore, a ZnO thin film was fabricated onto a PET substrate using low-pressure, high-frequency Ar + O_2_ plasma and a chemical vapor deposition technique to apply to DSSC devices. To achieve excellent electrical properties, high transmission, and efficiency of DSSC, the experimental conditions were compared by varying the RF power supply to 200, 220, 240, 260, 280, and 300 W.

## 2. Materials and Methods

### 2.1. Preparation of ZnO/PET Substrates

**Figure 1:**
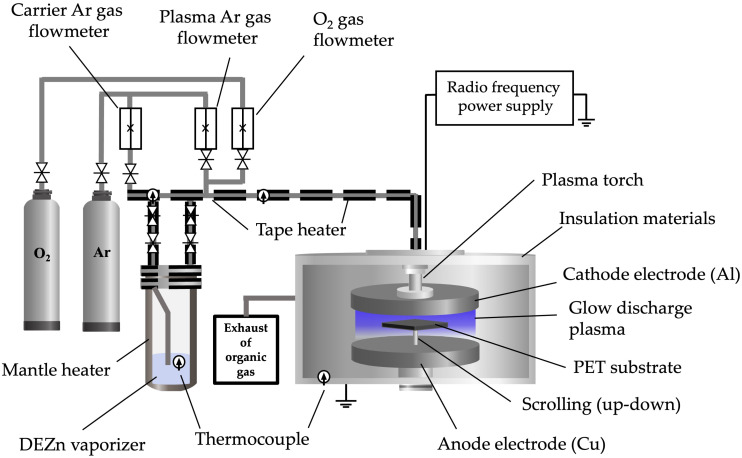
Equipment used in the low-pressure, high-frequency Ar + O_2_ plasma using chemical vapor deposition technique of ZnO film fabrication on PET substrates.

The fabrication system of the low-pressure, high-frequency plasma generator is illustrated in Figure 1. The plasma generator comprises an Al cathode and a grounded anode of Cu plate. A reactor machine of AYUMI INDUSTRY Co., Ltd., Himeji, Japan, used a PET substrate with a cooling system placed on top of the anode plate. A slit-type hole (11 mm) was made in the cathode hole (105 mm) to let Ar + O_2_ into the gap between the cathode and the anode. The anode plate scrolls with a PET substrate up and down in a direction perpendicular to the slit to produce flat films in a large area (scrolling distance: 25 mm). Atmospheric low-pressure high-frequency plasma was generated by flowing Ar as an insert gas with O_2_ [34,35,36,37]. The reaction of O_2_ and Ar ions (Equation (1)) is assumed to be the same as for the mutual neutralization of oxygen ions [38,39].

(1) $$O^{-}+{Ar}^{+}\to O+Ar$$

The Ar carrier gas was fed through the inner space of the cathode down to the gap, where it was stimulated by the RF plasma generator (AX-3000, AD-TEC Plasma Technology Co., Ltd., Hiroshima, Japan). Diethylzinc (C_2_H_5_)_2_Zn, or DEZn (Tri Chemical Laboratories Inc., Yamanashi, Japan), was vaporized and carried by the Ar + O_2_ carrier gas flow into the plasma generated in the gap. When DEZn comes into contact with air, it burns to form zinc oxide (ZnO), carbon dioxide (CO_2_), and water (H_2_O). The reaction occurs when DEZn comes into contact with oxygen molecules. The balanced chemical equation is as follows:

(2) $$Zn{{\left(C_{2}H_{5}\right)}_{2}}_{\left(s\right)}+{7O_{2}}_{\left(g\right)}\to {ZnO}_{\left(s\right)}+4{{CO}_{2}}_{\left(g\right)}+5{H_{2}O}_{\left(g\right)}$$

To allow the chemical reaction that forms a thin film on the substrate surface to proceed at an acceptable rate, it is necessary to remove product gases from the deposition environment. A vacuum pump (EBARA DRY PUMP A30W, Ebara Co., Tokyo, Japan) was used, which corresponds to the reaction flow and chemical properties of the gases. The parameters are listed in Table 1 according to the fabrication conditions for the film preparation.

### 2.2. Fabrication of DSSCs

The 1.5 cm × 1.5 cm ITO glass (Luminescence Technology Corporation, New Taipei, Taiwan) with a sheet resistance of 9 to 15 Ω/sq applied as the photoanode substrate. The ZnO/PET was coated on the ITO glass to form a film using a screen printing technique. It was then immersed in a D102 dye solution (95% HPCL, Sigma-Aldrich, Inc., Missouri, United States) at room temperature for 12 h to absorb dye molecules. After that, the films were rinsed with ethanol and dried. The DSSCs fabricated in the form of sandwiching on ITO-based and ZnO/PET with an electrolyte 0.2 M PMII, 0.05 M I2, 0.1 M LiI, 0.2 M TBAI, and 0.5 M TBP in AN/VN [10], as well as a thick thermal-plastic Surlyn spacer (SX1170-25, Solaronix S.A., Aubonne, Switzerland), were used.

### 2.3. Characterization

To investigate the ZnO thin film properties, Hall effect measurement (ECOPIA HMS-2000, Ecopia Corporation, Republic of Korea) and a four-point probe device (DASOL ENG FPP-HS-8, Dasoleng company, Chungcheong Buk-do, Republic of Korea) were used to measure the resistivity, carrier concentration, and mobility. A surface profilometer measured the film’s thickness (Dektak 150, Veeco Instruments Inc., Tokyo, Japan). A UV—visible spectrophotometer (U-3000, Hitachi, Ltd., Tokyo, Japan) was applied to analyze the optical transmittance spectra of ZnO thin films. Furthermore, the morphology of ZnO thin films deposited on the PET substrate was examined through the X-ray diffraction (XRD) (Rigaku D/max 2100H, Rigaku Holdings Corporation, Tokyo, Japan) technique. A field emission scanning electron microscope (FE-SEM: S-5500, Hitachi, Ltd., Tokyo, Japan) machine was used to observe the structural properties of the films with the atomic force microscope (AFM: Prak, X-100).

In addition, to analyze the elemental composition and chemical stage of ZnO/PET film surfaces prepared by low-pressure, high-frequency Ar + O_2_ plasma chemical vapor deposition technique, analysis data were obtained using the X-ray photoelectron spectroscopy (XPS) method by the KRATOS AXIS SUPRA (Shimadzu, Kratos Ultra2, Shimadzu Group Company, Kyoto, Japan) machine. Monochromatic Al k (1492.75 eV) X-rays were used at an emission of 10 mA and 15 kV HT (150 W) and an analysis area of 600 × 200. All data was recorded at a base pressure below 8 × 10^−9^ Torr and a temperature of 295 K.

## 3. Results

### 3.1. Physics of Surface Reaction under Low-Pressure, High-Frequency Ar + O_2_ Plasma of ZnO Film Fabrication on PET Substrate

**Figure 2:**
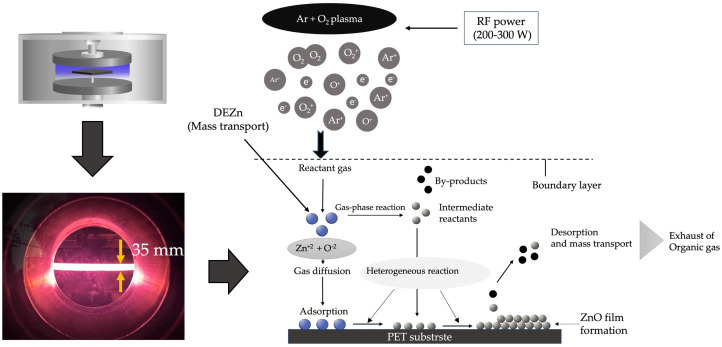
Reaction systems involving clusters of ZnO film formation under low-pressure, high-frequency plasma stimulation by low-pressure, high-frequency Ar + O_2_ plasma chemical vapor deposition on a PET substrate.

**Figure 3:**
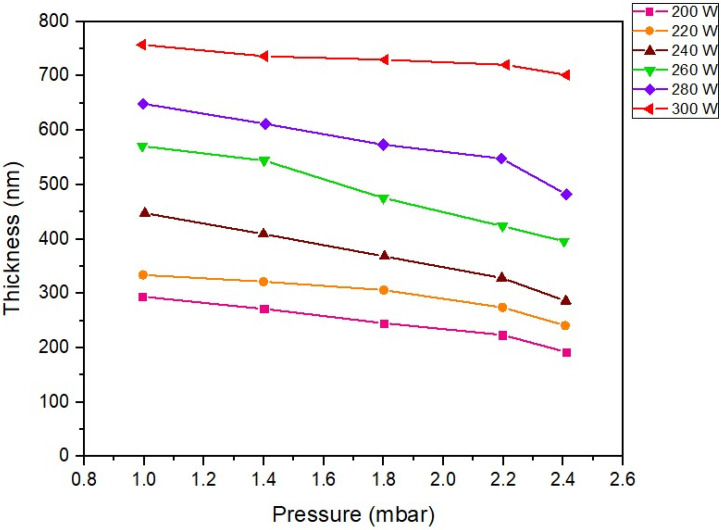
Influence of the variation RF power on ZnO film thickness prepared by low−pressure, high−frequency Ar + O_2_ plasma chemical vapor deposition technique.

In Figure 2, the process of chemical vapor deposition is illustrated. In the first stage, when precursor molecules of DEZn + O_2_ + Ar enter the reactor chamber, Zn^+2^ and O^−2^ molecules are transported to the surface of a PET substrate through diffusion [40] onto the substrate surface. After the heterogeneous reaction [41] on the surface, ZnO atoms remain. In contrast, the by-product molecules of the reaction on the substrate surface are adsorbed from the substrate, generating a free gap for more Zn^+2^ and O^−2^ molecules to enter. In this research, we drive the reaction by increasing energy in the process of the Ar + O_2_ plasma energy delivered to Zn^+2^ and O^−2^ molecules to increase the reaction rate to form a ZnO precursor on the substrate surface. Under these conditions, the growth rate of the thin film depends only on the rate of chemical reactions on the substrate surface through plasma energy activation. The growth rate of the ZnO thin film is presented in terms of thickness in Figure 3.

### 3.2. Influence of RF Power to the Thickness of ZnO Thin Films

Under low-pressure, high-frequency Ar + O_2_ plasma stimulated by gas phase collisions [42] with RF power significantly influences the delivery of ZnO to the substrate surface. The reason is that most reaction chemical species produced in the plasma result in a much higher surface reaction rate [43], resulting in differences in film thickness following different RF power supply activation conditions. In Figure 3, the thickness of the ZnO film layer at different fabrication conditions of excitation with an RF power range of 200–300 W is displayed. As can be seen in the graph, ZnO film prepared with an RF power of 300 W exhibits a maximum thickness greater than 700 nm. The reason is that the arrival rate of the ZnO precursor, also called precursor flux [44], is higher than that of the other excitation conditions of RF power. As a result, the adsorption rate of ZnO molecules on the substrate is significant and directly proportional to the power supply condition of 200–300 W. However, if reaction by-products (Figure 2) are still absorbed, they will prevent the arrival of the ZnO precursor, reducing the adsorption rate. The results can be observed at an RF power of 280–200 W, respectively, and increased pressure in the reactor chamber.

### 3.3. Structral Properties of ZnO Film Deposited on PET Substrates

**Figure 4:**
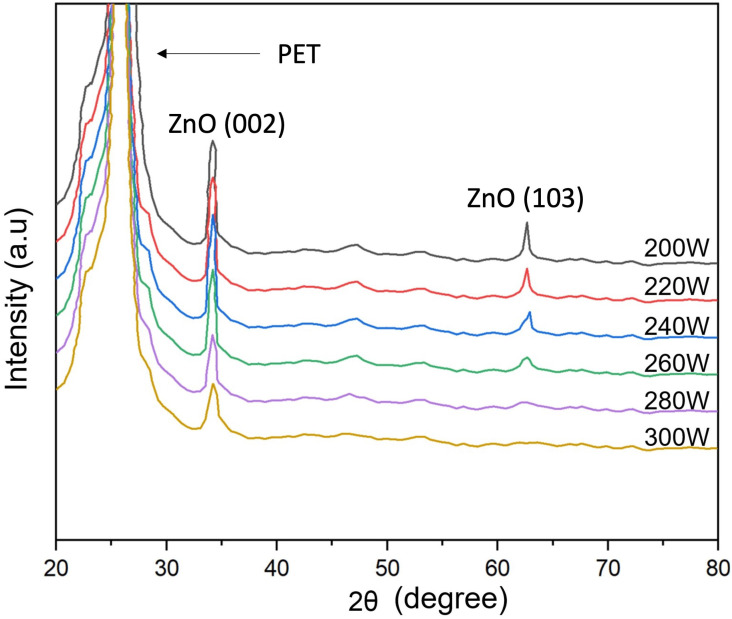
X-ray diffraction analysis (XRD) of ZnO thin film deposited on a PET substrate prepared by low-pressure, high-frequency Ar + O_2_ plasma chemical vapor deposition.

Figure 4 presents the XRD pattern of the ZnO film on the PET substrate under the diffraction pattern achieved with two from 20° to 80°. The information shows that the ZnO peaks detected are recognized as having a polycrystalline hexagonal wurtzite crystal structure. The XRD spectra of all samples display ZnO (002) located at approximately 34.24°. This result is consistent with the previous study [22,45,46]. Noticeably, the image information shows that as the RF power increased, the intensity of the diffraction peak (002) continuously rose, which increased the film thickness, consistent with Figure 3. The small, broad peak (103) is also observed. However, the diffraction intensity of (002) is more potent than that of the (103) direction. According to the effect of film deposition on the ZnO film thickness result in Figure 3, the results indicate that the ZnO thin film structure mainly appears with a columnar grain structure pattern perpendicular to the substrate surface. Therefore, the research results show that the film thickness can change the crystal orientation of the ZnO film formation, which is directly affected by the plasma simulation of increasing RF power during the deposition process. For this reason, a suitable film thickness is required to create highly oriented ZnO crystalline thin films. The average crystalline size (D) of the ZnO film is determined using Scherrer’s equation [47].

(3) $$D=\frac{k\lambda }{\beta \cos\theta }$$

**Figure 5:**
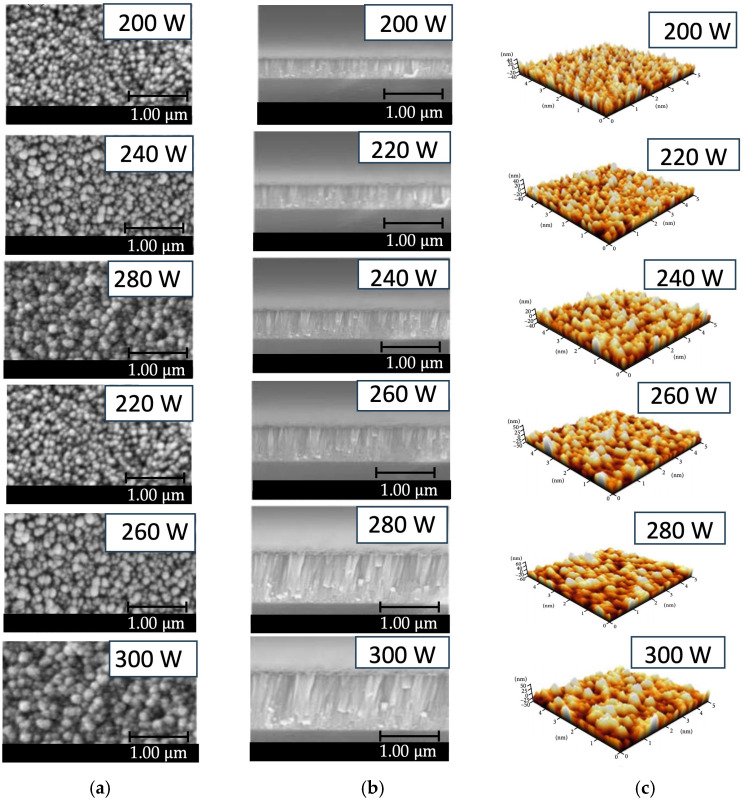
Microstructure image of the ZnO thin film deposited on PET substrate under different conditions of RF power range of 200–300 W prepared by low-pressure, high-frequency Ar + O_2_ plasma chemical vapor deposition: (**a**) FE-SEM images of top-view; (**b**) FE-SEM images of cross-section view; (**c**) AFM images.

Figure 5a,b illustrates FE-SEM images consisting of a top view (5a) and cross-section view (5b) of a ZnO thin film surface prepared with low-pressure, high-frequency Ar + O_2_ plasma chemical vapor deposition. The test results found that the ZnO film prepared with an RF power of 300 W had the characteristic of forming a dense and uniform columnar film with a thickness of 720 nm. The surface of the film had a granular structure. The grains are prominent in the 50–150 nm range, with an average of approximately 100 nm. According to the observation, plasma energy affects film formation. Where the RF power is low, the film is thin, the density is low, and the grains are small. When the RF power increases, the film has a dense columnar appearance. When it is more uniform, the film surface has a granular structure and the grain is larger and more defined. Thus, the FE-SEM images show that using low-pressure, high-frequency Ar + O_2_ plasma to stimulate the production of ZnO thin films significantly affects film growth. For films with higher RF power, which has a significant energy content, the kinetic energy of ZnO atoms will increase, and the diffusion coefficient will also be high. In this case, it moves throughout the available space, causing the density of large grains to increase on the film. On the other hand, while preparing films with lower RF power, the kinetic energy of the ZnO atoms was low. It has a low diffusion coefficient, resulting in scattered, sparse clumps, resulting in low deposition. It gives a columnar crystal structure with a low density. Additionally, Figure 5c displays an AFM image of a ZnO film showing film surface characteristics consistent with the FE-SEM images in Figure 5a,b.

**Figure 6:**
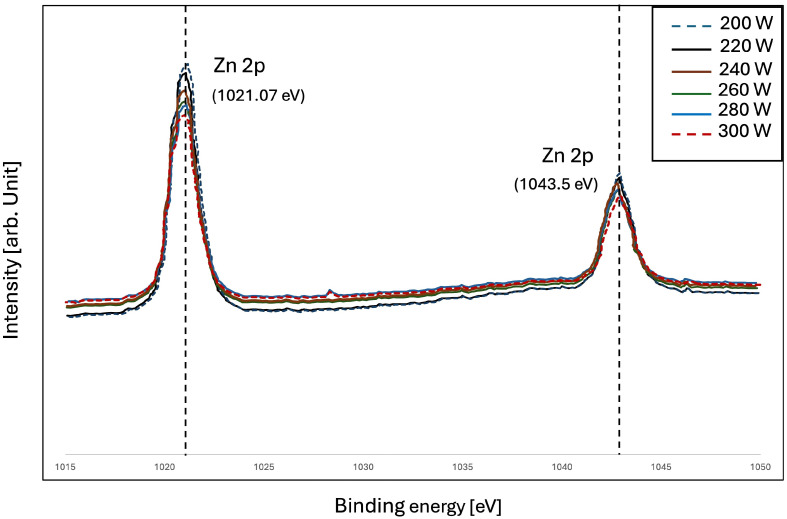
X-ray photoelectron spectroscopy spectra of ZnO thin films deposited on PET substrates with narrow scanning spectroscopy of Zn 2p.

**Figure 7:**
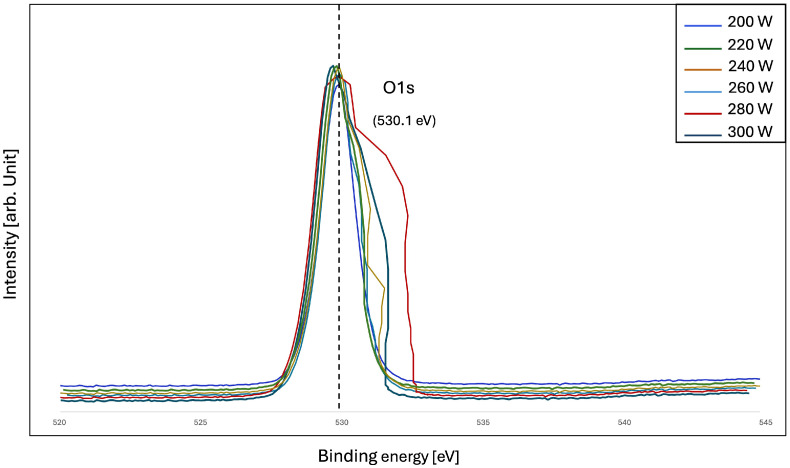
X-ray photoelectron spectroscopy spectra of ZnO thin films deposited on PET substrates with narrow scanning spectroscopy of O1s.

To further verify the above results, the bonding state on the surface of the ZnO/PET film was examined. The basic principles of XPS are as follows: When irradiated with X-rays, a photoelectric effect occurs. Photoelectrons are created from atoms in a sample by collecting photoelectrons with electrostatics and analyzing their energy with an electrostatic hemispherical analyzer. The XPS method makes it possible to determine the kinetic energy of the photoelectrons. Thus, the XPS spectrum of these photoelectrons is measured, and the binding energy between the atomic nucleus and the electron is determined based on the difference in the energy of the incident X-rays. Figure 6 and Figure 7 show the structural investigation results of ZnO/PET films compared in the wide-axis XPS spectrum. The surface composition and chemical state of ZnO films were investigated using the XPS method. In the XPS spectra, the binding energies of all elements were calibrated using the carbon C1s peak around 285.0 eV as a reference. The Zn 2p core electrons for ZnO/PET films prepared with an RF power range of 200–300 W are at the same level with two binding energies at 1021.07 eV and 1043.5 eV, as shown in the figure. At the same time, the O1s peak at about 530.1 eV, located in the O group in the Zn-O of framework for both films, has a shoulder occurred at higher binding energy. This shoulder peak is related to the oxygen defect sites related to the oxygen atom vacancies or chemisorbed oxygen in the ZnO framework [48,49].

### 3.4. Electrical Properties

**Figure 8:**
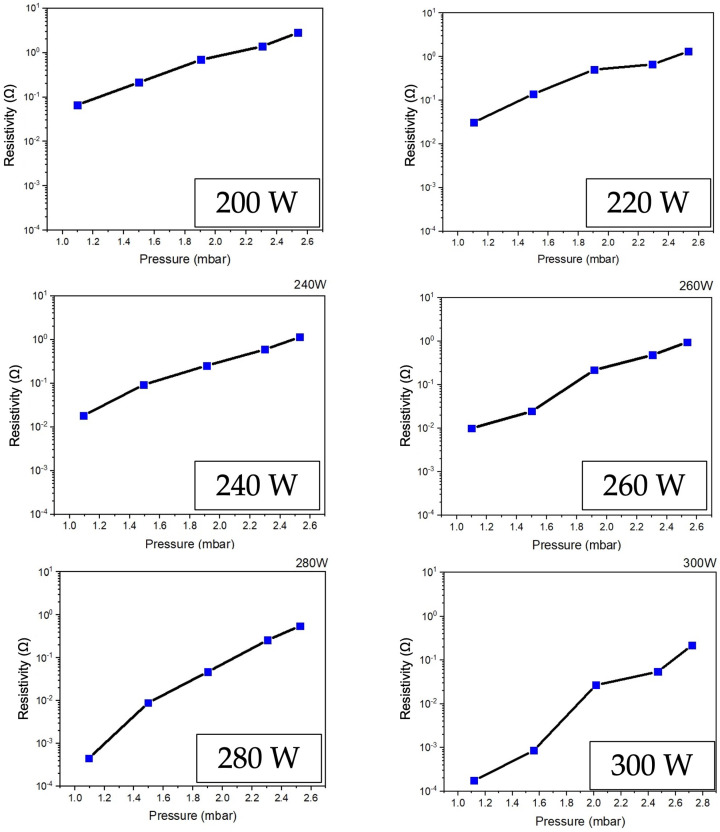
Electrical resistivity of ZnO thin film deposited on PET substrate comparing by different low-pressure, high-frequency Ar + O_2_ plasma supply with an RF power range of 200–300 W.

Figure 8 illustrates the comparative results of the electrical resistivity of ZnO films prepared with different plasma power excitation conditions at an RF power range of 200–300 W. The trend of the graph shows that it is related to the ZnO film thickness investigation results in Figure 3, which has the lowest electrical resistivity value of 1.8 × 10^−4^ Ω under the condition of film preparation at an RF power of 300 W. This is because when stimulating the film depositing process with higher plasma energy, it will support the reaction between the precursors. Thus, the final product will be thicker ZnO films with a smooth texture and consistency. 

**Figure 9:**
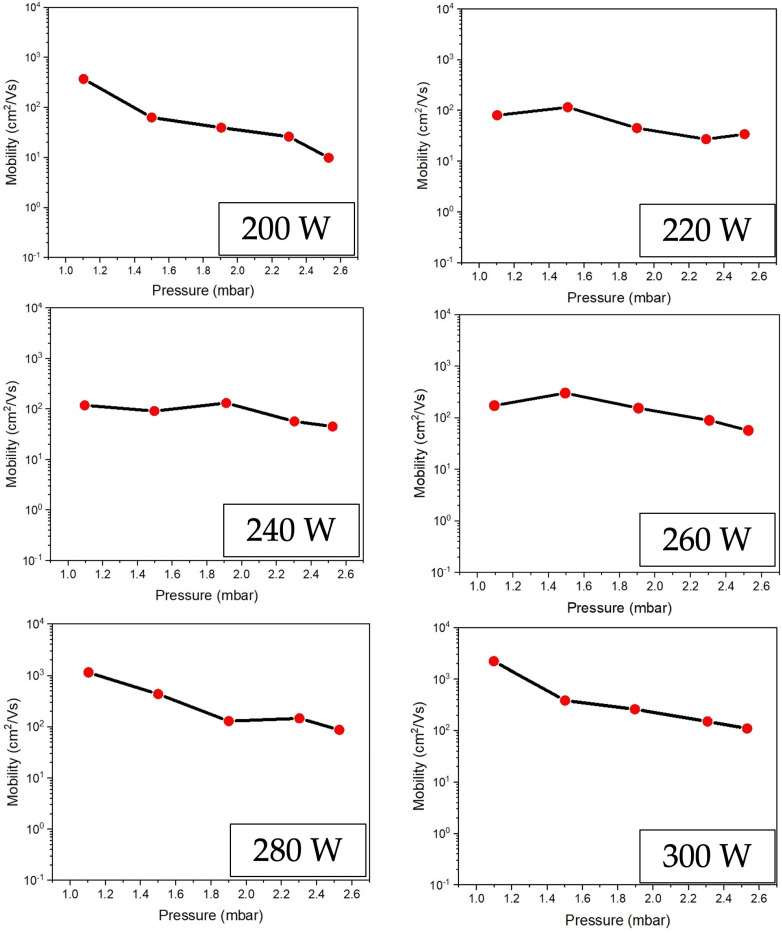
Comparison the carrier mobility on ZnO thin film preparing at different conditions with an RF power range of 200–300 W under low-pressure, high-frequency Ar + O_2_ plasma of chemical vapor deposition.

**Figure 10:**
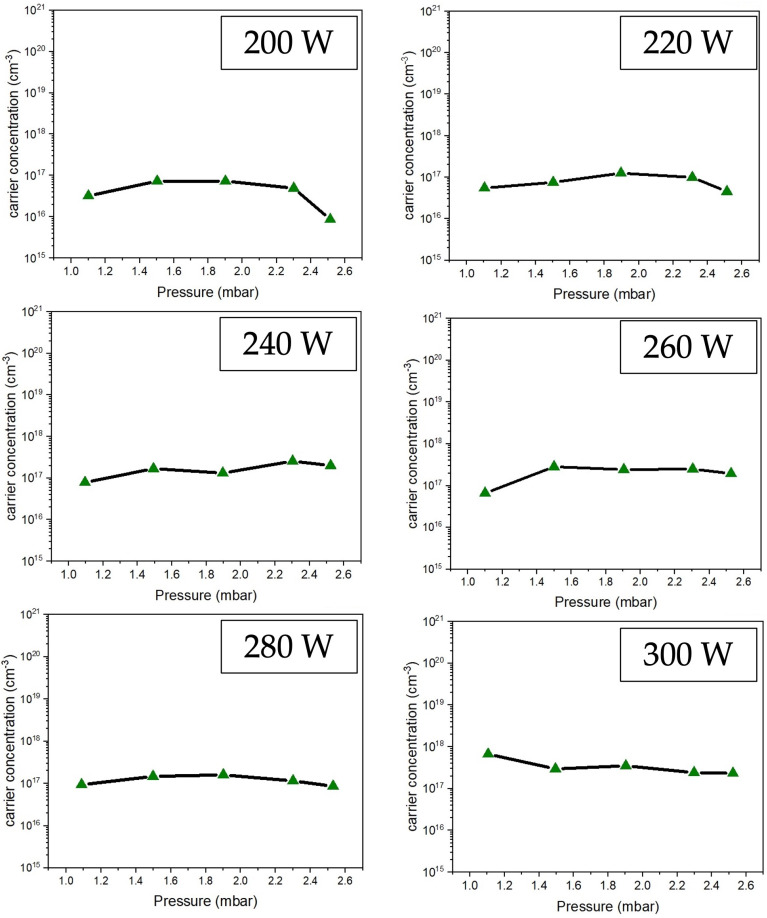
Comparison the carrier concentration on ZnO thin film preparing at different conditions of an RF power range of 200–300 W under low-pressure, high-frequency Ar + O_2_ plasma of chemical vapor deposition.

Figure 9 compares carrier mobility and different conditions of RF power supply to ZnO fabrication. The results indicate that carrier mobility declines as RF power increases. It is inversely proportional to the electrical resistivity graph, as shown in Figure 8. It can be assumed that low electrical resistivity is shown when the carrier mobility is high. Figure 10 displays the effect of RF power on the carrier concentration. The results showed similar values, but 300 W gives a maximum carrier concentration of about 9.7 × 10^17^ cm^−3^. Table 2 summarizes the result in each RF power supply case to make it easier to understand.

### 3.5. Transmittance Properties

**Figure 11:**
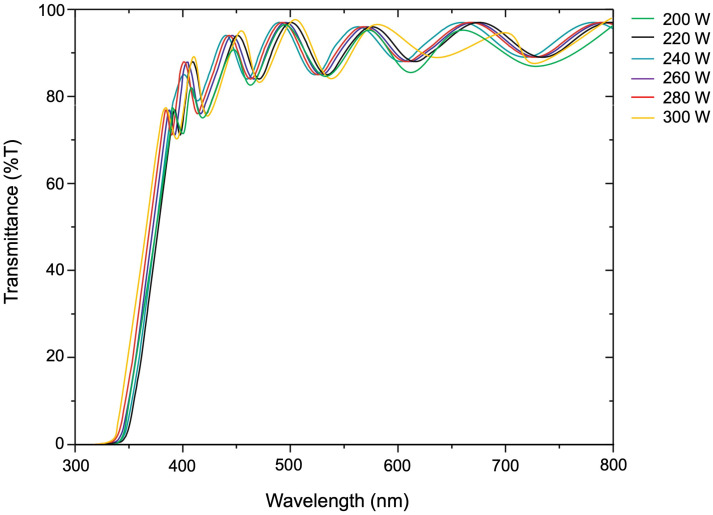
Light transmittance of ZnO thin film deposited on PET substrate preparing by low-pressure, high-frequency Ar + O_2_ plasma chemical vapor deposition under different conditions with an RF power range of 200–300 W.

The light transmittance of the ZnO film was measured using a dual-beam UV–visible spectrometer, as shown in Figure 11. The results showed that all samples had light transmission in the wavelength range of 400–800 nm. Due to the thickness of the films, the interference streaks that occurred were sine waves. When measuring the light transmittance, it appears as a precise sine wave. The average light transmittance percentage increased as the film thickness decreased. The reaction on the substrate surface in Figure 1 explains the reason. Due to the lower plasma energy excited, the ZnO precursor is less likely to fall onto the substrate. As a result, the thickness of ZnO films will be less, increasing light transmittance.

### 3.6. Photovoltaic Characterization of DSSCs

**Figure 12:**
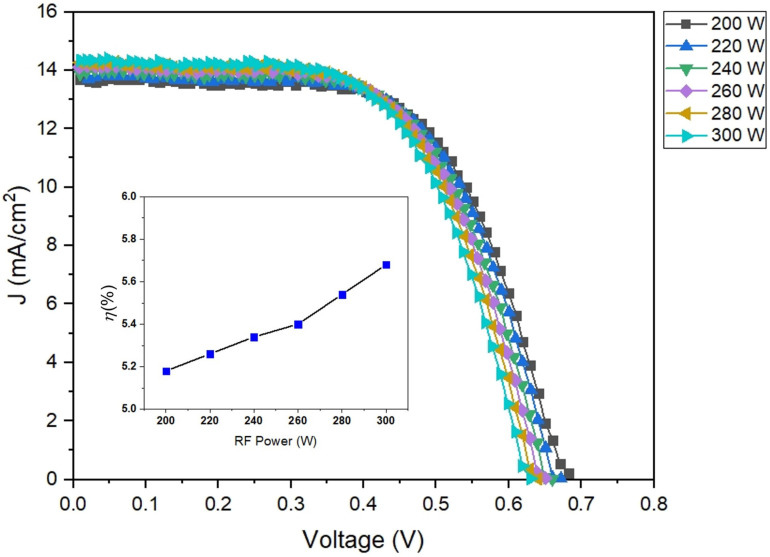
Photocurrent density-photovoltage (J–V) curves of DSSCs based on ZnO/PET substrates.

In this work, the parameters were determined to calculate the power conversion efficiency (PEC), which includes short circuit current density ($J_{sc}$); open circuit voltage ($V_{oc})$; maximum current density ($J_{max})$, which is the maximum electric current per area while the voltage is equal to zero; maximum potential ($V_{max}$) is the voltage value when is no current or equal to zero; and fill factor (FF), which is the ratio between the multiple of maximum current density and maximum potential divided by the multiple of short circuit current density and open circuit voltage [50,51], as seen in the photocurrent density–photovoltage (J–V) curves of DSSCs in Figure 12. Therefore, a power conversion efficiency (PCE) can be calculated by the multiple of $V_{oc}$, $J_{sc}$, and FF divided by an incident light power ($P_{in}$) [48,49] as shown in Table 3, Equation (4).

(4) $$PCE\left(\eta \right)=\frac{\left(V_{oc}\times J_{sc}\times FF\right)}{P_{in}}$$

Figure 12 presents the power conversion efficiency of DSSCs based on ZnO/PET substrate by one sun illumination of AM 1.5 G with 100 mW/cm^2^. The DSSCs with a ZnO/PET and deposition conditions with an RF power of 300 W show a higher short circuit current density of 14.32 mA/cm^2^ and improved FF value of 0.63 with an open-circuit voltage of 0.63 V in comparison with the other samples, which results in a high power conversion efficiency of 5.68%.

## 4. Discussion

The research found that a ZnO thin film was fabricated using low-pressure, high-frequency Ar + O_2_ plasma and the chemical vapor deposition technique from DEZn under different film formation conditions. This will produce films with different electrical properties, optical properties, and film surface characteristics. The results can be summarized as follows:

Firstly, in thin film production, ZnO precursors generated by RF plasma excitation are adsorbed on the surface of a PET substrate. After that, decomposition will occur under heterogeneous reactions through interaction with the substrate surface. In this case, the DEZn, O_2_, and Ar molecules excited by RF power decompose to form ZnO atoms on the surface of the substrate, and Ar and O_2_ molecules are absorbed into the gas phase. The rate of degradation of Ar and O_2_ depends on the temperature and vacuum pressure in the film formation process. Thus, the surface reactions that create ZnO thin films in these deposition processes consume energy from high-energy chemicals produced using plasma, an RF plasma source. The reason is that free ions and electrons in the plasma can attack the growing film directly.

Second, when considering the RF power and vacuum pressure, also consider the effect on the deposition and thickness of the ZnO films. As the pressure increased, the film thickness tended to decrease. This is because the number of argon ions in plasma generation will increase. When the vacuum pressure increases, the ZnO atoms collide with the argon ions in the plasma simulation system in the reaction chamber and then lose energy due to the collision, causing them not to have enough power to drop on the substrate. In this case, the deposition rate will decrease. Meanwhile, ZnO film prepared with higher RF power resulted in a corresponding increase in film layer thickness. Due to the energy supply, increasing plasma production accelerates chemical reactions, resulting in high-energy collisions and momentum transfers between atoms. As a result, the atoms have a high energy of movement while depositing onto the substrate. In this case, a thicker film will be obtained compared to the sample prepared with a low power.

Finally, vacuum pressure and RF power affect the film’s electrical resistivity and physical characteristics. As the vacuum pressure in the system increases, the electrical resistivity increases. As the RF power increases, the electrical resistance decreases. For this, both the carrier nobility and carrier density influence the electrical resistivity of the film. The increasing vacuum pressure in the system produces more positive ions in the plasma. In this, when positive ions collide with ZnO atoms, they have a chance of losing energy from colliding between ions, causing the kinetic energy of ZnO atoms to be reduced, with a low diffusion coefficient leading them to clump together into scattered clusters. These will result in a low deposition rate and a columnar crystal structure with a low density, resulting in a film with high electrical resistivity. However, adding plasma energy to the system through RF power will stimulate atoms with more deposition energy to have more kinetic energy and a high diffusion coefficient, creating a thick film layer and large grains.

## 5. Conclusions

The ZnO thin films are entirely fabricated by low-pressure, high-frequency Ar + O_2_ plasma using a chemical vapor deposition technique. Among the comparisons of each sample under different RF power supplies (200, 220, 240, 260, 280, and 300 W), an RF power of 300 W exhibited the lowest electrical resistivity of 1.8 × 10^−4^ Ω, which is the most comfortable property to apply in a DSSC device. At this point, the efficiency measurement is presented at 5.68%. Therefore, the results will be helpful in the solar cell manufacturing industry and provide a new way to create ZnO thin films for use as solar cells.

## Figures and Tables

**Table 1:** Fabrication conditions of ZnO thin films deposited on PET substrate by low-pressure, high-frequency plasma deposition technique.

Source and Materials	Conditions
Substrate	PET
Source material	DEZn of Tri Chemical Laboratories Inc.
Carrier Ar gas flow rate	20 L/min
O_2_ gas flow rate	0.5 L/min
RF plasma generator	200–300 W
Scrolling distance	25 mm
Anode and cathode gap	35 mm

**Table 2:** The comparison of electrical properties on ZnO thin films deposited on PET substrates including resistivity, mobility, and carrier concentration under different RF power supplies.

RF Power (W)	Resistivity (Ω)		Mobility (cm^2^/V·s)		Carrier Concentration (cm^−3^)	
	Lowest	Highest	Lowest	Highest	Lowest	Highest
200	7.2 × 10^−2^	1.7	1.2 × 10^1^	3.1 × 10^2^	7.6 × 10^15^	1.5 × 10^16^
220	2.2 × 10^−2^	1.1	4.1 × 10^1^	1.1 × 10^2^	3.2 × 10^16^	8.5 × 10^16^
240	1.7 × 10^−2^	7.3 × 10^−1^	4.6	1.8 × 10^2^	9.7 × 10^16^	2.3 × 10^17^
260	1.2 × 10^−2^	9.2 × 10^−1^	7.4	1.2 × 10^2^	8.3 × 10^16^	2.2 × 10^17^
280	5.1 × 10^−4^	6.4 × 10^−1^	9.8	1.2 × 10^3^	8.9 × 10^16^	1.7 × 10^17^
300 *	1.8 × 10^−4^	3.1 × 10^−1^	1.2 × 10^2^	2.1 × 10^3^	2.3 × 10^17^	9.7 × 10^17^

* This is a greatest result.

**Table 3:** Photovoltaic parameters of DSSCs based on the ZnO/PET substrates of varying RF power between 200 and 300 W.

ZnO/PET Substrate	J_sc_ (mA/cm^−2^)	V_oc_ (V)	FF	Efficiency (η, %)
RF 200 W	13.66	0.69	0.55	5.18
RF 220 W	13.78	0.67	0.57	5.26
RF 240 W	13.95	0.66	0.58	5.34
RF 260 W	14.10	0.65	0.59	5.40
RF 280 W	14.21	0.64	0.61	5.54
RF 300 W	14.32	0.63	0.63	5.68

## Data Availability

The original contributions presented in the study are included in the article, further inquiries can be directed to the corresponding author.
